# The Nature and the Mode of Propagation of Phthisis

**Published:** 1967-07

**Authors:** William Budd

**Affiliations:** Consulting Physician to the Bristol Royal Infirmary


*We print below the announcement in the Lancet of 12th October, 1867, to which Dr. Wofinden refers in the preceding article—Editors.*

MEMORANDUM ON

### THE NATURE AND THE MODE OF PROPAGATION OF PHTHISIS

BY WILLIAM BUDD, M.D.

*Consulting Physician to the Bristol Royal Infirmary.*

---

*"He that would follow philosophy must be a freeman in mind."*—PTOLEMY.

---

[NOTE FROM DR. PAGET.]

*To the Editor of* THE LANCET.

SIR,—The paper I send enclosed was received by me last December, in a sealed packet, from Dr. William Budd, of Clifton, with a request that I would take charge of it until he should direct me to break the seal. At his desire, I opened the packet a few days ago, and I now send you the contents, requesting the favour of their early publication in THE LANCET. They are an epitome of what Dr. W. Budd has been for some time intending to publish in a more complete form; but his intention has been frustrated, and is still delayed, by the engrossments of professional practice and other circumstances beyond his control.

You will at once perceive the originality of his views, and their very high importance if established. If the evidence now given of their truth be incomplete, it is at least abundantly sufficient to raise them out of the region of mere hypothesis, and ensure their careful consideration by pathologists.

In a letter to me Dr. W. Budd says he can show strong reasons for believing that his views on tubercle, with certain qualifications, apply to cancer also.

I am, &c.,

Cambridge, Sept. 30th, 1867. G. E. PAGET.

---

THE following are the principal conclusions to which I have been led regarding Phthisis or Tubercle :—

1st. That tubercle is a true zymotic disease, of specific nature, in the same sense as typhoid fever, scarlet fever, typhus, syphilis, &c., &c., are.

2nd. That, like these diseases, tubercle never originates spontaneously, but is perpetuated solely by the law of continuous succession.

3rd. That the tuberculous matter itself is (or includes) the specific morbific matter of the disease, and constitutes the material by which phthisis is propagated from one person to another, and disseminated through society.

4th. That the deposits of this matter are, therefore, of the nature of an eruption, and bear the same relation to the disease, phthisis, as the " yellow matter ' of typhoid fever, for instance, bears to typhoid fever.

5th. That, by the destruction of this matter on its issue from the body, by means of proper chemicals or otherwise—seconded by good sanitary conditions,—there is reason to hope that we may, eventually, and possibly at no very distant time, rid ourselves entirely of this fatal scourge.

The evidence on which these conclusions are founded is drawn from the following principal sources:—

(a) Considerations based on the pathology of phthisis, as showing it to consist in the evolution and multiplication within the organism of a specific morbid matter, with a universal tendency to elimination and casting forth of the same, after the type of zymotic diseases generally.

(b) Actual instances in which there was evidence to show that phthisis was communicated from one person to another.

(c) The geographical distribution of phthisis in past and present times, and, especially, its great fatality now in countries which when first discovered by Europeans were known to be entirely free from it.

(d) Its much greater prevalence in low levels and among crowded communities, and its entire absence, unless by casual importation, at very high levels,—conditions which are well known to rule, in the same directions, the spread of zymotic diseases generally, and especially of that group in which, as in phthisis, the morbific matter is cast off in a liquid form.

(e) Its very high rate of prevalence in convents, harems, barracks, penitentiaries, &c.—that is to say, under the very social conditions which are known most to favour the propagation of diseases of the zymotic group.

Among the data relating to geographical distribution the following striking facts may be here mentioned:—

1st. When the South Sea Islands were first discovered phthisis did not exist there. Since the aborigines have come into intimate contact with Europeans the disease has not only made its appearance among them, but has become so widespread as to threaten their extermination.

The contrast between original entire immunity and present extreme fatality is very striking, and can only be rationally explained by the importation of a new and specific morbific germ.

Try every other supposition, and the facts are inexplicable; make this one supposition, and they are at once explained.

2nd. The late Dr. Rush, of Philadelphia, who made very accurate inquiries to determine the point, satisfied himself that when America was first discovered, phthisis was unknown among the native American Indians. Now it is very fatal to them.

The very significant contrast here exhibited between the past and present history of these two races, in respect of phthisis, is exhibited at once, and at the present time, among the negro race in Africa, in different parts of the area of that great continent.

It is well known that negroes are peculiarly liable to phthisis.

Now, everywhere along the African seaboard where the blacks have come into constant and intimate relations with the whites, phthisis causes a large mortality among them. In the interior, where intercourse with the whites has been limited to casual contact with a few great travellers or other adventurous visitors, there is reason to believe that phthisis does not exist. Dr. Livingstone and other African travellers have given me the most positive assurances on this point.

The idea that phthisis is a self-propagated zymotic disease, and that all the leading phenomena of its distribution may be explained by supposing that it is disseminated through society by specific germs contained in the tuberculous matter cast off by persons already suffering from the disease, first came into my mind, unbidden, so to speak, while I was walking on the Observatory hill at Clifton, in the second week of August, 1856. The close analogy in many quite fundamental points between this disease and typhoid fever had often impressed itself on me with very great force while I was engaged in the study of the latter, and in the preparation of the papers which I have published on it. I now saw with a clearness which had never occurred to me before, that, with the exception of the qualifications necessary for their application to a chronic disease—for the most part of slow evolution and indefinite duration,—the leading conclusions to which I had been led respecting the propagation of the fever, might be applied with the same strictness to phthisis also.

This idea had no sooner taken possession of my mind than considerations of great force and in overwhelming number crowded upon me in illustration of it.

In the course of the same evening I drew up some notes on the subject, and before the end of the month my views upon it had taken, in outline, the exact shape which they now have. The long interval which has occurred between the summer of 1856 and the present date has been occupied in collecting data bearing on the various questions raised by this new theory—in accumulating evidence of various kinds, and in examining and carefully weighing difficulties. During the whole of this long time the subject has scarcely ever been absent from my mind. The result has been only to confirm me more and more in the truth of my first conclusions. I earnestly hope that they will not be lightly rejected. At any rate, I can say that they have not been brought forward in haste, or without due deliberation. I have, in fact, considerably exceeded the ten years, which, with a fine sense of what is due to such an enterprise, the Roman poet prescribed as the time to be given to every composition intended by the writer to endure.

Many causes have helped to prevent me from giving my views on this subject sooner to the world. Chief among them I may name want of time to put them into that scientific form, and clear logical order, under which alone an innovation so daring has any chance of being entertained, much more of being accepted, by the profession. This task, however, I hope to complete in the course of a few months. Meanwhile I have thought it well to place this memorandum, by way of record, in the hands of a friend, to be made public at any moment should occasion seem to require it.

Manor House, Clifton, Dec. 1st, 1866.

